# Accuracy of intracranial pressure monitoring: systematic review and meta-analysis

**DOI:** 10.1186/s13054-015-1137-9

**Published:** 2015-12-02

**Authors:** Lucia Zacchetti, Sandra Magnoni, Federica Di Corte, Elisa R. Zanier, Nino Stocchetti

**Affiliations:** Department of Physiopathology and Transplant, Milan University, Via Festa del Perdono 7, 20122 Milan, Italy; Department of Anesthesiology and Intensive Care, Fondazione IRCCS, Cà Granda-Ospedale Maggiore Policlinico, Via F. Sforza 35, 20122 Milan, Italy; IRCCS Istituto Mario Negri, Department of Neuroscience, Via G. La Masa 19, 20156 Milan, Italy

## Abstract

**Introduction:**

Intracranial pressure (ICP) measurement is used to tailor interventions and to assist in formulating the prognosis for traumatic brain injury patients. Accurate data are therefore essential. The aim of this study was to verify the accuracy of ICP monitoring systems on the basis of a literature review.

**Methods:**

A PubMed search was conducted from 1982 to 2014, plus additional references from the selected papers. Accuracy was defined as the degree of correspondence between the pressure read by the catheter and a reference “real” ICP measurement. Studies comparing simultaneous readings from at least two catheters were included. Drift was defined as the loss of accuracy over the monitoring period. Meta-analyses of data from the studies were used to estimate the overall mean difference between simultaneous ICP measurements and their variability. Individual studies were weighted using both a fixed and a random effects model.

**Results:**

Of 163 articles screened, 83 compared two intracranial catheters: 64 reported accuracy and 37 drift (some reported both). Of these, 10 and 17, respectively, fulfilled the inclusion criteria for accuracy and zero drift analysis. The combined mean differences between probes were 1.5 mmHg (95 % confidence interval (CI) 0.7–2.3) with the random effects model and 1.6 mmHg (95 % CI 1.3–1.9) with the fixed effects model. The reported mean drift over a long observation period was 0.75 mmHg. No relation was found with the duration of monitoring or differences between various probes.

**Conclusions:**

This study confirms that the average error between ICP measures is clinically negligible. The random effects model, however, indicates that a high percentage of readings may vary over a wide range, with clinical implications both for future comparison studies and for daily care.

## Introduction

Intracranial pressure (ICP) monitoring is widely used in neurointensive care, especially for the management of patients with traumatic brain injury (TBI). ICP levels are used to decide interventions, to verify the efficacy of therapeutic maneuvers, and in formulating a prognosis. For all these purposes accurate measurements are essential.

Clinicians rely on numbers provided by different methods, and generally believe that those numbers reflect actual ICP with a high degree of accuracy. For instance, a recent trial on decompressive craniectomy randomized patients to different treatments when ICP exceeded 20 mmHg [1], assuming that the sensors used ensured a clear-cut threshold.

In real life, however, ICP measurement is challenging. A basic assumption is that the ICP read at one point in the intracranial system properly reflects the average pressure throughout all other locations. Under normal conditions the cerebrospinal fluid (CSF) pressure should in fact be in equilibrium in the whole of the CSF space. Under pathological conditions (as when CSF circulation is obstructed or intracranial masses are expanding), however, ICP cones can develop leading to uneven ICP levels [2, 3]. Even when ICP is homogeneous in the whole intracranial space measurements may not all be accurate, depending on the devices used and the duration of the measurement [4–6].

Our hypothesis is that clinically used systems measure ICP with different degrees of accuracy, not always providing a precise measure. Therefore, our goal was to evaluate the accuracy and precision over time (drift) of different ICP measurements on the basis of a review of the literature.

## Methods

### Literature search

A PubMed (http://www.ncbi.nlm.nih.gov/pubmed) search was made, looking for studies published from January 1982 through November 2014. The following keywords were searched: “intracranial pressure” AND “Camino”, “intracranial pressure” AND “Codman”, “intracranial pressure” AND “fluid filled”, “intracranial pressure” AND “tip transducer”, “intracranial pressure” AND “drift”. The search was limited to articles in English and animal studies were included at this stage. Additional articles were screened through the references listed in the papers initially selected.

### Accuracy

Accuracy was defined as the degree of correspondence between the pressure read by the catheter and a reference “real” ICP measurement. In order to assess accuracy in vivo*,* studies comparing simultaneous readings from at least two catheters were included, divided into two groups:

- Group 1: studies comparing a ventricular fluid-coupled catheter with external transducer (VFC) with another type of device. According to the guidelines of the Brain Trauma Foundation [7], this is the most accurate method for measuring ICP so we took it as the reference standard.
- Group 2: studies comparing catheters other than VFC.

Some articles included substudies with different sets of paired readings from probes. These were analyzed as separate studies.

Studies on intracranial probes located in the epidural, subdural, or subarachnoid space or comparing infratentorial versus supratentorial probes were excluded from the analysis. Studies were screened for numerical data, and only those reporting the limits of agreement and bias (or data from which they could be calculated) were included. Animal studies were not included in this analysis.

### Zero drift

Drift was defined as the loss of accuracy over the monitoring time, a problem related to catheters such as intraparenchymal probes that cannot be re-zeroed during monitoring. In the selected papers drift was assessed by two methods: a) verifying the pressure read by the catheter at atmospheric pressure once it was removed from the patient [8, 9]; and b) measuring pressure changes over time in an artificial hydraulic model [10, 11]. Animal studies were included in this analysis.

### Statistics

**Fig. 1:**
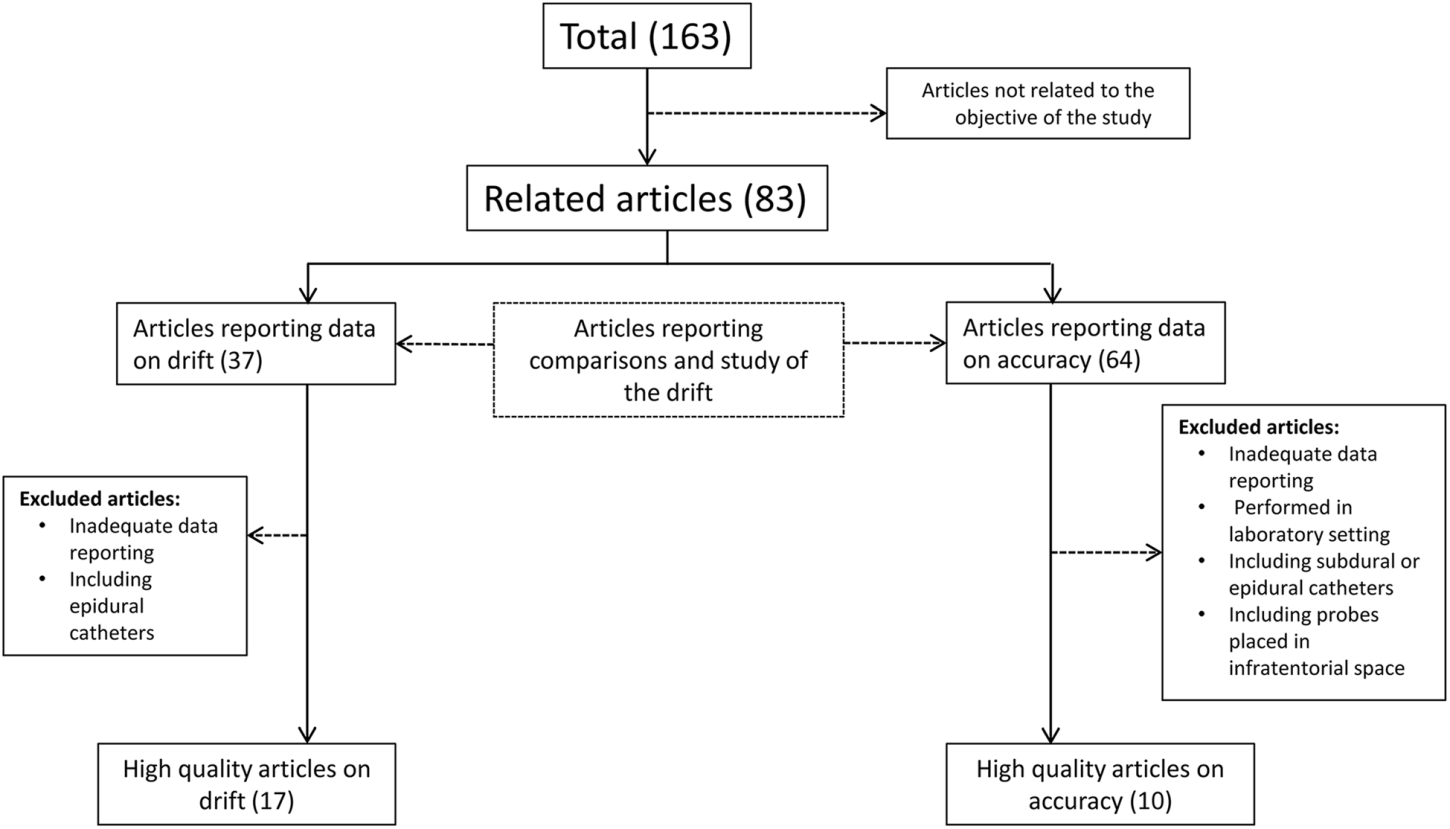
Flow diagram summarizing article selection; 83 articles were identified:64 reported accuracy, 37 reported drift (some reported both). Articles on animals, with probes placed in the epidural, subdural, or infratentorial spaces and with inadequate data reporting were excluded. High-quality articles on drift reported the range, mean and median of drift; the 17 papers refer to 20 studies. High-quality articles on accuracy included comparisons of two ICP probes, with Bland-Altman analysis and adequate data reporting; the 10 papers listed refer to 15 studies

Mean ICP differences between the various probes, standard deviation and limits of agreement were reported in most papers. If these summary measures were not available, but mean ICP readings per patient were presented, as read with different probes, we calculated the mean ICP differences and standard deviations (Table 1). Studies for which the results were only given in plots or summary tables were not suitable for the planned analysis, and therefore they were not included on the basis of inadequate data reporting (Fig. 1). The authors of five relevant papers reporting incomplete information [12–16] were asked to provide the original data, but this was not accessible (mainly because it was collected decades ago), and these studies were not included either.

**Table 1:** Main features of the studies included in the meta-analysis

	Author/year of publication	Probe 1	Probe 2	Probe placement	Patients (n)	Calibration/zeroing^a^	Mean difference	Standard deviation	Correlation	Data^b^
Group 1										
1	Schickner 1992 [4]	Camino	VFC	P–V	10	+/+	9.20	7.80	NA	Reported
2	Chambers 1993 [21]	Camino	VFC	V–V	10	+/+ ^(EAM)^	1.43	2.26	0.98	Calculated
3	Statham 1993 [22]	Camino	VFC	P–V	11	+/+	2.73	4.22	0.98	Reported
4	Gopinath 1995 [23]	Codman	VFC	V–V	25	+/+	0.50	2.60	0.97	Reported
5	Signorini 1998-2 [24]	Codman	VFC	P–V	2	+/+ ^(EAM)^	1.81	3.75	NA	Calculated
6	Chambers 2001 [25]	Spiegelberg	VFC	P–V	11	+/+ ^(EAM)^	0.10	4.99	NA	Reported
7	Koskinen 2005 [8]	Codman	VFC	P–V	22	+/+ ^(EAM)^	1.20	3.32	0.79	Reported
8	Lescot 2011-1 [26]	Pressio	VFC	P–V	15	+/+ ^(EAM)^	–0.60	3.83	NA	Reported
9	Lescot 2011-2 [26]	Codman	VFC	P–V	15	+/+ ^(EAM)^	0.30	3.52	NA	Reported
10	Eide 2012-2 [18]	Codman	VFC	P–V	5	NA/NA	4.52	13.97	NA	Calculated
Group 2										
11	Signorini 1998-1 [24]	Codman	Camino	P–P	5	+	1.58	3.36	NA	Calculated
12	Sahuquillo 1999-1 [2]	Camino	Camino	P–P	33	NA	1.80	1.10	0.95	Reported
13	Sahuquillo 1999-2 [2]	Camino	Camino	P–P	16	NA	7.50	6.40	0.85	Reported
14	Eide 2012-1 [18]	Codman	Codman	P–P	5	NA	0.64	5.59	NA	Calculated
15	Eide 2012-3 [18]	Codman	Spiegelberg	P–P	7	+	0.70	2.63	NA	Calculated

Studies comparing simultaneous readings from at least two catheters for intracranial pressure monitoring classified depending on the reported use (Group 1) or not use (Group 2) of a reference standard (i.e.VFC) and reported in chronological order. Some papers reported more than one study; four are cited repeatedly. In two studies a single catheter was placed in the ventricles (V-V). This gave a typical reading through a fluid-filled system and a simultaneous measurement through a solid transducer at the tip

^a^Description of calibration (of the solid probe) and zeroing (of the ventricular fluid-filled catheter). (+) Calibration or zeroing is reported, (++) both calibration and zeroing are reported; *NA* information on calibration and zeroing maneuvers not available, ^*(EAM)*^ zeroing described at the external auditory meatus

^b^Indicates how the mean intracranial pressure differences between probes and standard deviations were determined (see Methods)

*NA* Not Available, *P* Parenchymal, *V* Ventricular, *VFC* Ventricular Fluid-Coupled catheter with external transducer

The limits of agreement between simultaneous ICP measurements were calculated using the following formula:

$$ \overline{x}\pm {t}_{\left(0.025,n-1\right)}s\sqrt{1+\frac{1}{n}} $$

where $\overline{x}$ equals the mean difference, *s* the standard deviation of the differences, and *t*(0.025, *n*–1) the value from Student’s *t*-distribution corresponding to a two-sided alpha of 0.05 and (n–1) degrees of freedom. While still based on the assumption of normal distribution of probe differences around a mean (“bias” or “offset”), this method compensates for the size of the sample, includes an adjustment for the expected differences in the mean, and it is therefore more widely applicable than the large sample approximation of ($\overline{x}$ ± 1.96 *s*). 

Meta-analyses of data from the studies were performed to calculate the overall mean difference between simultaneous ICP measurements and the variability. In calculating the overall mean difference across the combined studies, results from individual studies were weighted, assigning the weights using two standard approaches: a fixed effects model and a random effects model.

A fixed effects approach assumes there is one true mean ICP difference between paired probe values. The overall mean difference is calculated by weighting individual study results by the inverse of the observed variance. The random effects approach assumes that the true mean ICP difference between paired probes can vary from one study to another. The overall mean difference is calculated by weighting individual studies according to within-study and between-study variability.

Summary statistical analysis was done using the software program SPSS (Version 21, IBM/SPSS, Inc.) and for meta-analyses we used the software package Comprehensive Meta Analysis (Version 2.2.064, Biostat, Inc.).

## Results

The literature search identified 163 studies; 80 were excluded because they were not related to the aim of this study. Of the remaining 83, only 64 reported comparisons of two paired intracranial catheters (Fig. 1).

### Accuracy

After studies based on catheters placed in the epidural space, or comparing the infratentorial and supratentorial spaces, or with inadequate data reporting were excluded, 10 articles were left for accuracy analysis (Fig. 1). They reported 15 studies, 10 in Group 1, and 5 in Group 2. The main features of the studies are reported in Table 1, including details on calibration of solid probes before insertion, and zeroing of ventricular fluid-filled catheters. Calibration and/or VFC zeroing maneuvers were reported in the vast majority of Group 1 studies, whereas in Group 2 these maneuvers were less accurately or not reported at all. ICP recordings for individual studies covered a wide range (up to 100 mmHg) and all studies included cases with ICP >20 mmHg. Limits of agreement for individual studies are summarized in Table 2.

**Table 2:** Limits of agreement of the studies included in the meta-analysis

	Author and year of publication	Patients (n)	Lower limit of agreement	Mean difference	Upper limit of agreement
Group 1					
1	Schickner 1992 [4]	10	−9.31	9.20	27.71
2	Chambers 1993 [21]	10	−3.92	1.43	6.78
3	Statham 1993 [22]	11	−7.09	2.73	12.55
4	Gopinath 1995 [23]	25	−4.97	0.50	5.97
5	Signorini 1998-2 [24]	5	−9.58	1.81	13.21
6	Chambers 2001 [25]	11	−11.51	0.10	11.71
7	Koskinen 2005 [8]	22	−5.86	1.20	8.26
8	Lescot 2011-1 [26]	15	−9.08	–0.60	7.88
9	Lescot 2011-2 [26]	15	−7.50	0.30	8.10
10	Eide 2012-2 [18]	5	−37.97	4.52	47.01
Group 2					
11	Signorini 1998-1 [24]	5	−8.65	1.58	11.82
12	Sahuquillo 1999-1 [2]	33	–0.47	1.80	4.07
13	Sahuquillo 1999-2 [2]	16	−6.56	7.50	21.56
14	Eide 2012-1 [18]	5	−16.37	0.64	17.65
15	Eide 2012-3 [18]	7	−6.17	0.70	7.57

**Fig. 2:**
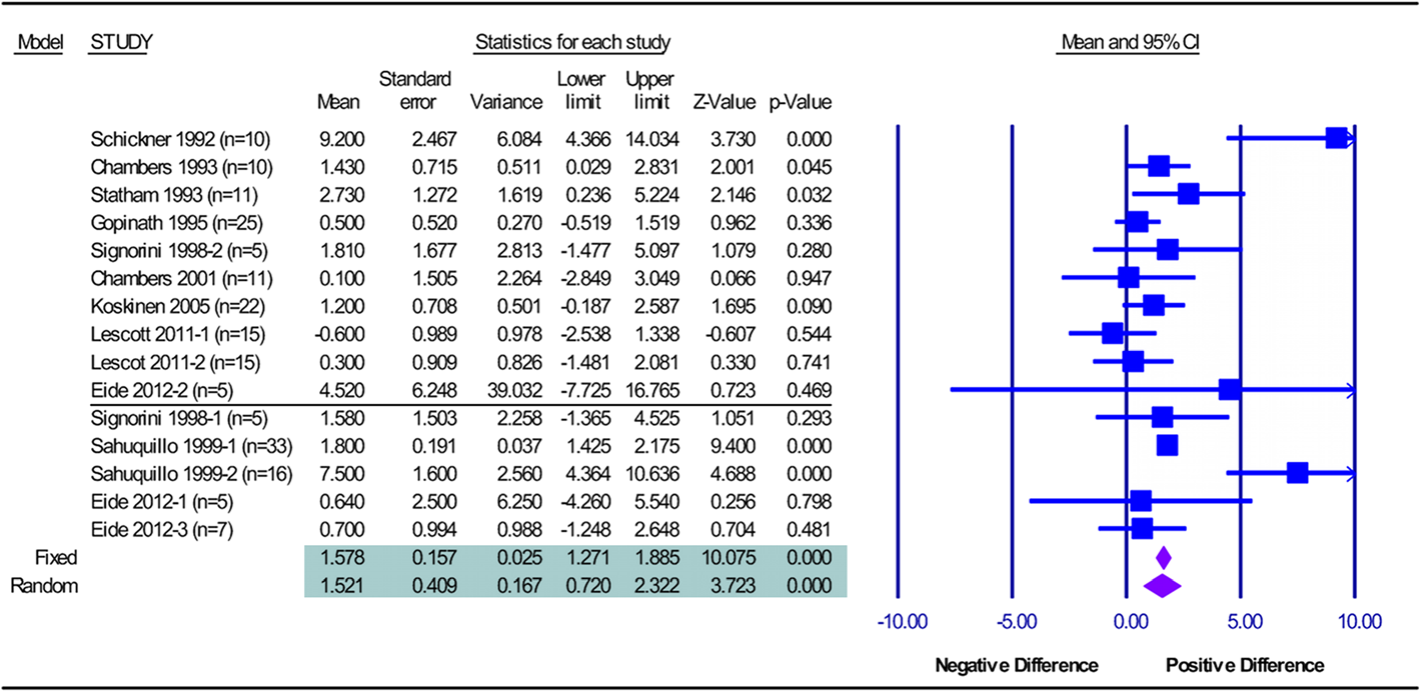
Meta-analysis. Meta-analysis data with the corresponding forest plot for individual data sets (combined Groups 1 and 2). The black horizontal line divides Group 1 (above) from Group 2 (below) studies, as reported in methods. *CI* Confidence Interval

Both positive and negative differences between probes were observed in individual studies. The combined estimate is a positive difference for probe 1 minus probe 2 readings, partly because of the ordering of the probes in the calculation. All comparisons of the two locations in Group 1 were standardized so that the difference was always parenchymal–ventricular, leaving only one study with negative differences (Table 1). For other paired readings between catheters in similar locations (parenchymal–parenchymal or ventricular–ventricular), the catheter with the highest average reading was identified as probe 1, so as to give consistent positive (and not offsetting) mean differences. The combined mean difference between probes, using a fixed model,was 0.9 mmHg, with a 95 % confidence interval (CI) for the mean of 0.4–1.5 mmHg across Group 1 studies and 1.8 mmHg (95 % CI 1.5–2.2 mmHg) across Group 2 studies. The combined mean difference between all probes, assuming the fixed model, was 1.6 mmHg (95 % CI 1.3–1.9 mmHg) (Fig. 2).

The combined mean difference between probes, using a random model, was 1.2 mmHg (95 % CI 0.2–2.1 mmHg) in Group 1 studies and 2.3 mmHg (95 % CI 0.5–4.2 mmHg) in Group 2 studies. The combined mean difference between all probes, pooling all studies and assuming the random effects model, was 1.5 mmHg (95 % CI 0.7–2.3 mmHg) (Fig. 2).

### Zero drift

The zero drift analysis was reported in 37 articles but only 17 had adequate data reporting (Table 3). Although some papers reported a wide range of values, the mean drift over a long observation period was 0.75 mmHg.

**Table 3:** Main features of the studies reporting drift

	Author and year of publication	Probe type	Readings (n)^a^	Test duration (days)	ICP range (mmHg)
Clinical studies					
1	Statham 1993 [22]	Fiberoptic	11	1–11	0/+4
2	Bavetta 1997 [27]	Fiberoptic	83	1–12	−12/+14
3	Münch1998 [28]	Fiberoptic	95	1–17	−15/+12
4	Martines-Mañas 2000 [29]	Fiberoptic	56	1–12	−24/+35
5	Poca 2002 [19]	Fiberoptic	126	1–11	−12/+7
6	Stendel 2003-1 [17]	Fiberoptic	50	1–32	0/+12
7	Gelabert-González 2006 [9]	Fiberoptic	624	1–5	−17/+21
8	Gopinath 1995 [23]	Microstrain gauge	25	2–7	−2/+2
9	Stendel 2003-2 [17]	Microstrain gauge	98	3–28	−2/+3
10	Koskinen 2005 [8]	Microstrain gauge	128	1–16	−5/+4
11	Citerio 2008 [30]	Microstrain gauge	89	1–10	−4/+8
12	Al-Tamimi 2009 [20]	Microstrain gauge	88	3; 6^b^	NA
13	Lang 2003 [31]	Microstrain gauge	84	3–28	−2/+2
Laboratory studies					
14	Czosnyka 1996-1 [32]	Fiberoptic	1	3	−0.8/+0.8
15	Czosnyka 1996-2 [32]	Fiberoptic	1	3	−0.4/+0.4
16	Piper 2001 [33]	Fiberoptic	34	1–12	−13/+22
17	Sundbӓrg 1987 [12]	Microstrain gauge	1	3	−2/+2
18	Czosnyka 1996-3 [32]	Microstrain gauge	1	3	−0.8/+0.8
19	Morgalla 1999 [10]	Microstrain gauge	7	10	−4/+3
20	Citerio 2004 [11]	Microstrain gauge	10	5	0/+2

Studies reporting drift were classified according to the setting (clinical or laboratory). Some papers reported more than one study; one paper is cited three times. Since different probes were analyzed in some studies, a single reference may appear more than once

^a^Indicates how many readings of drift were used to calculate the range, mean and/or median (see text for more details)

^b^The study by Al-Tamimi et al. included data from two centers and reported the median observation time for both

*ICP* Intracranial pressure, *NA* Not available

No differences were detected between fiberoptic probes and microstrain-gauge probes (mean fiberoptic 0.59 ± 1.8; mean microstrain-gauge 0.95 ± 0.23; *p* = 0.8), or between data from clinical and laboratory studies (clinical mean 0.81 ± 0.83; laboratory mean 0.48 ± 0.63; *p* = 0.82).

Eleven papers addressed the degree of drift as a function of the duration of use; ten found no correlation while one [17] described a weak positive correlation (Spearman’s correlation coefficient 0.342; *p* = 0.001).

## Discussion

This review found that when two ICP measurements were taken simultaneously using different sensors the averages were close, but with a large standard deviation.

The 10 studies in Group 1 compared various sensors to ventricular catheters. Ventricular catheters are connected through a fluid-filled system (VFC) to an external transducer, which can be zeroed at will, offering a reliable reference standard. The calculation of the average ICP difference in Group 1 included both positive and negative values, which may have reduced the final picture (the absolute difference).

While the majority of studies indicated only small differences between the intraparenchymal probe and the VFC, two reported widely differing ICP levels. Schickner et al. [4] studied 10 patients with refractory ICP. They used a Camino fiberoptic probe, and a ventricular catheter was inserted to drain CSF. The Camino readings, on average, were 9 mmHg higher than the VFC, with single episodes of 40 mmHg difference. Eide et al. [18] retrospectively studied patients after subarachnoid or intraparenchymal bleeding. In five cases a Codman catheter was compared with simultaneous readings by VFC; differences were greater than 10 mmHg in three cases, and one single case had large differences in all measurements, with negative ICP. The authors suggested that significant human errors in zeroing, or sensor damage, may have occurred.

Group 2 comprised five studies comparing parenchymal probes without a ventricular catheter as a standard reference. Often these papers were published in order to prove the existence of ICP gradients, and it is therefore not surprising that they found a striking mean difference [2]. Additionally, in this group all differences were positive, so there were no negative values to offset the final average. This may explain why the mean differences were slightly larger, but still only around 1–2 mmHg.

To sum up all the studies, we used both a fixed model and a random model. Although all the studies involved a comparison of simultaneous ICP measurements, the populations were not homogeneous. They reflected a cross-section of clinical cases, with differences in the manufacturer and type of sensor used, the sensor placement, and the type and extent of brain injury. Since all these factors can potentially affect ICP measurements, it cannot be assumed that all the studies were investigating an unknown but constant difference between paired ICP readings. We therefore feel that the assumptions for the random effects model are more appropriate. Nonetheless, the results with both models were closely comparable.

According to the random model, the combined mean difference between probes was 1.5 mmHg, with a 95 % CI of 0.7–2.3 mmHg. The simple pooled estimate of the standard deviation (unweighted) across the 15 data sets was 4.4 mmHg. Based on this estimate, a future study (similar to the average composition of the 15 studies examined) comparing two simultaneous ICP readings would be expected to have 95 % of the observed differences in the interval 1.5 ± (2 × 4.4) mmHg. Using the random standard error from the meta-analysis, which includes between-study variability, this interval would be slightly higher at 1.5 ± (2 × 5.7) mmHg. According to the random effects model, 70 % of readings could therefore vary in the range of ±6 mmHg, and 95 % of readings in the range of ±11.4 mmHg.

Discrepancies can be “true”, when there are pressure differences in the various areas of the brain, or due to inaccurate readings. True differences, when intracranial gradients are caused by expanding masses or unilateral hemorrhages, call for clinical judgment. In clinical routine, ICP is monitored with a single catheter in the overwhelming majority of patients. In case of unilateral masses with large shift, pressure gradients ( with higher pressure on the lesion side) are possible. In this situation the ICP data have to be interpreted considering where the catheter is placed. Inaccurate readings due to technical problems are more worrying since they are not due to actual intracranial gradients (and cannot be suspected in the computed tomography scan) but to erroneous measurement. Judging from the results of the meta-analysis, true discrepancies are relatively rare, but may be significant, up to ±11.4 mmHg. Clinicians should always carefully analyze the monitored data and also look at trends, possibly in the context of multimodal monitoring.

Fluid-coupled catheters can be re-zeroed as often as necessary while other sensors, in which zeroing cannot be repeated after insertion, may become less precise over time. Zero drift has been reported for several catheters under clinical and laboratory conditions [8–11, 19]. We found the mean drift was limited (on average less than 1 mmHg); however, single catheters varied widely. In general the pressures were within the limits specified by the manufacturers. There seem to be no significant differences between the various probes in clinical and experimental studies. All papers but one [20] showed no clear correlation between drift and length of ICP monitoring. We can therefore assume that drift is not related to the duration of monitoring, as previously indicated [7].

Our review, pooling different studies often with different designs, has limitations. The bulk of evidence on this topic is also fairly old: the median year of publication of studies on accuracy was 1998, and the range 1992–2012; for studies on drift the median year of publication was 2001 (range 1987–2009). The standards for publication have improved with time, with the result that recent studies provided more comprehensive information compared to the old ones.

## Conclusions

The clinical implications of our findings may be relevant both for future comparisons and for daily care. ICP monitoring is widely used to guide diagnostic and therapeutic decisions, so it is essential to know how reliable are the current techniques. The latest update of the Brain Trauma Foundation guidelines [7] concluded that parenchymal transducer devices measure ICP similarly to VFC but have the potential for differences. Our findings confirm that the average error is clinically negligible, but the interval of error is wide.

## Key messages

- A systematic literature review and meta-analysis indicates that the average error between simultaneous ICP measures is small (in the order of 1.5–1.6 mmHg), but in 30 % of readings it could exceed ±6 mmHg
- When there are intracranial gradients due to expanding masses or unilateral hemorrhages, there are likely to be larger differences between separate ICP measures.
- In the absence of intracranial gradients, the risk of inaccurate measurement seems rare.
- These findings are important both for future comparisons studies and for daily care.

## Abbreviations

CI: Confidence interval
CSF: Cerebrospinal fluid
ICP: Intracranial pressure
TBI: Traumatic brain injury
VFC: Ventricular fluid-coupled catheter with external transducer
